# Supplementary figures and images for: Predicting inmate suicidal behavior with an interpretable ensemble machine learning approach in smart prisons
Source: PeerJ Comput Sci. 2024 Jun 19;10:e2051. doi: 10.7717/peerj-cs.2051 (PMC11232594; doi:10.7717/peerj-cs.2051)

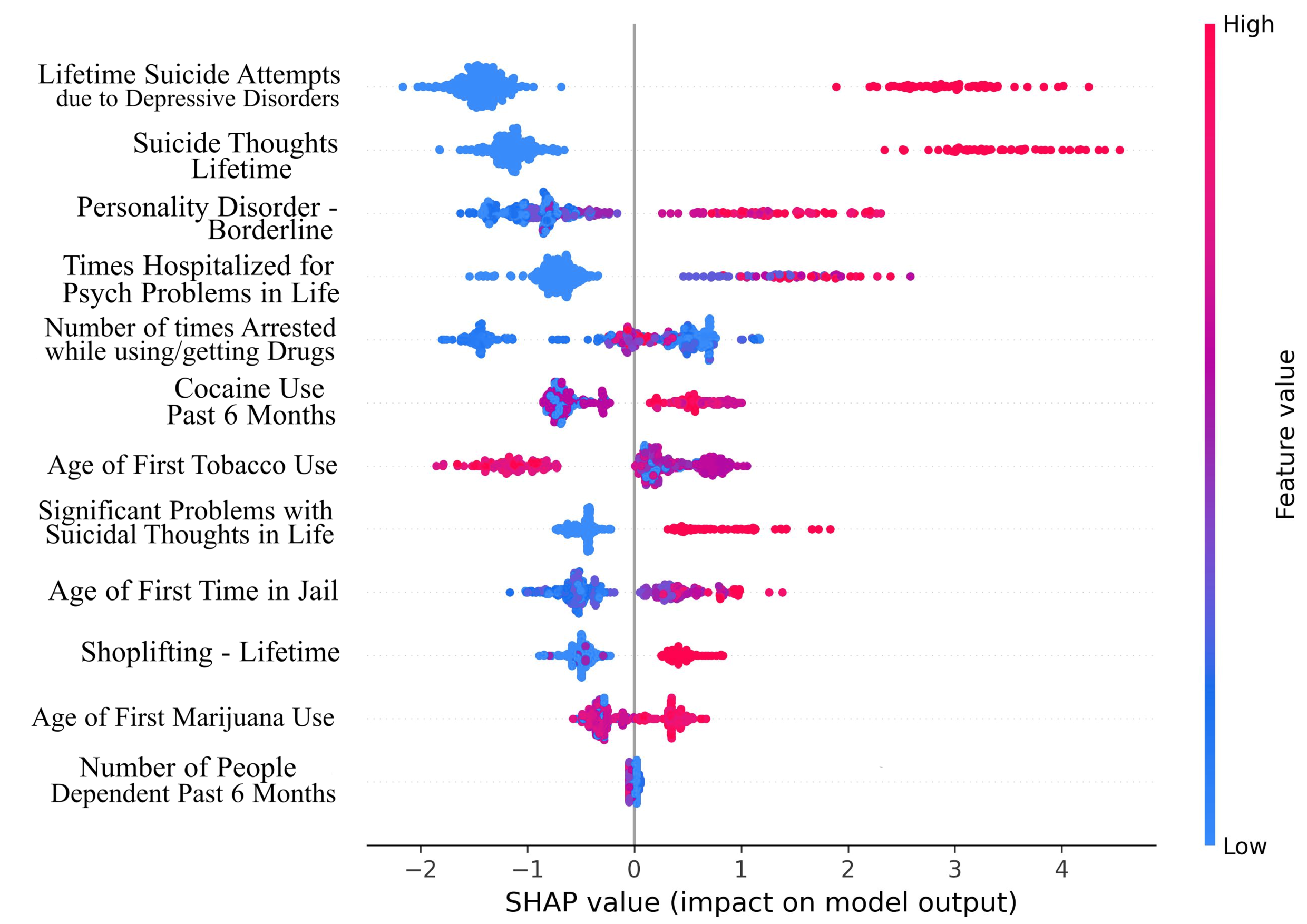

Supplement: Supplemental Information 1 [file peerj-cs-10-2051-s001.png]

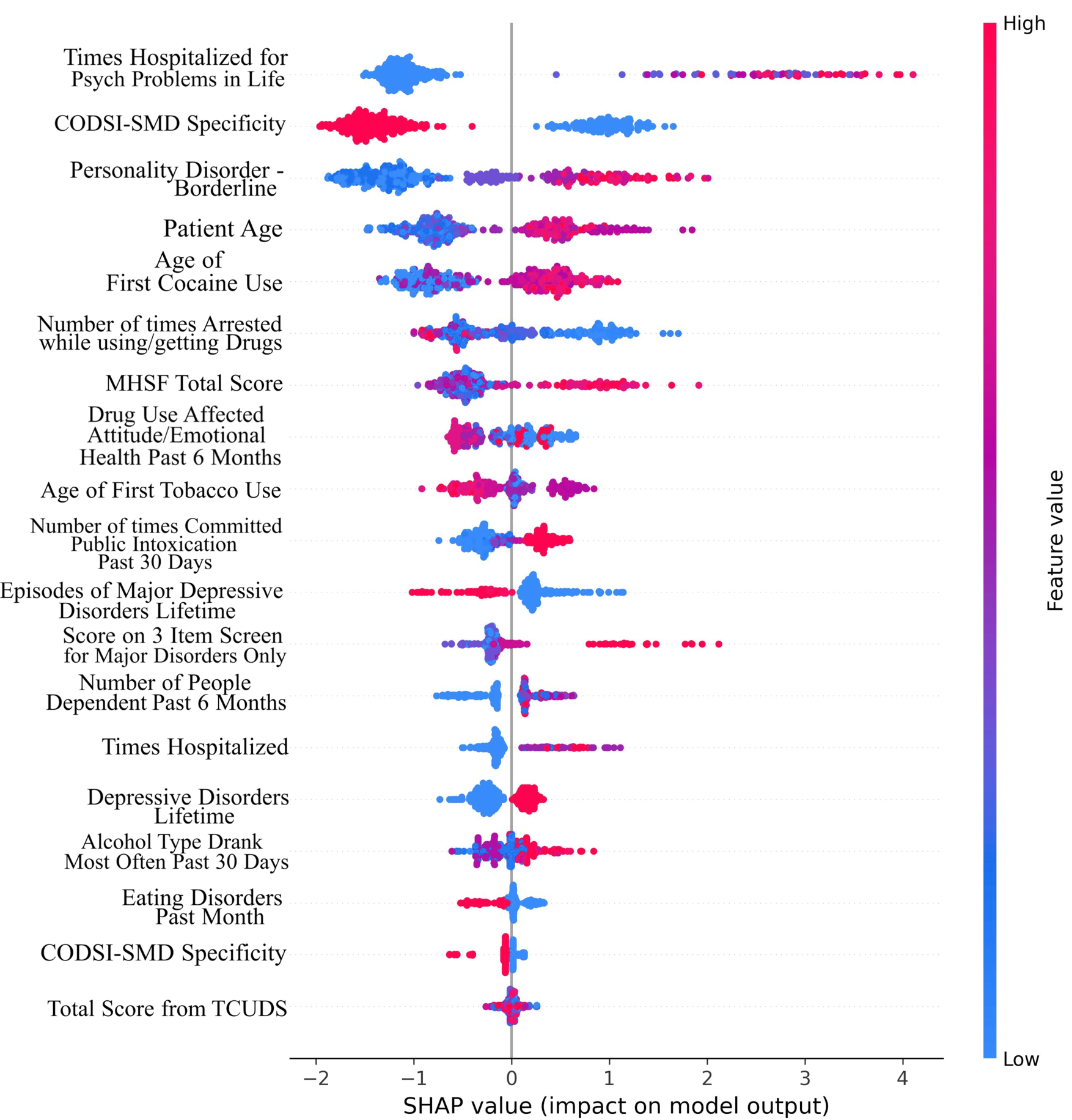

Supplement: Supplemental Information 2 [file peerj-cs-10-2051-s002.png]

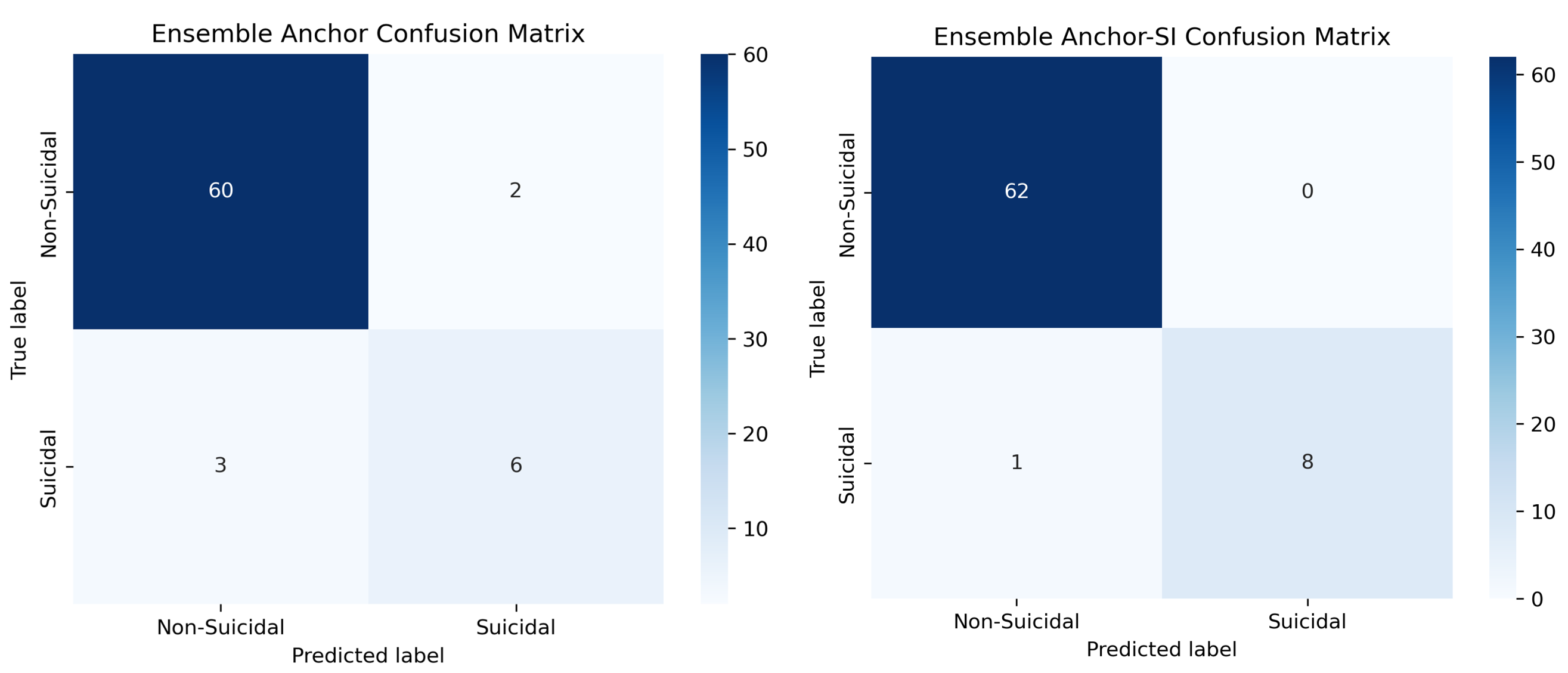

Supplement: Supplemental Information 3 [file peerj-cs-10-2051-s003.png]
