# Supplementary material for: Predicting inmate suicidal behavior with an interpretable ensemble machine learning approach in smart prisons
Source: PeerJ Comput Sci. 2024 Jun 19;10:e2051. doi: 10.7717/peerj-cs.2051 (PMC11232594; doi:10.7717/peerj-cs.2051)
Supplement: Supplemental Information 7 [file peerj-cs-10-2051-s007.pdf]

1 Supplementary Material

The supplementary material presented herein accompanies the main manuscript titled "Predicting Inmate Suicidal Behavior with an Interpretable Ensemble Machine Learning Approach in Smart Prisons" for PeerJ. This document aims to provide readers with additional details and visualizations that complement the findings discussed in the main paper.

Algorithm 1 Algorithm to obtain top 27 features using SHAP on XGBoost

```
procedure SHAP(Suicide_D, NonSuicide_D, X)
  XGB ← XGBClassifier
  DS ← Suicide_D, NonSuicide_D
  for datasets D in DS do
    T, S ← 80_20_split(D)
    XGB ← Fit(T)
    XGB ← Pred(S)
    xFrame ← DataFrame(X, X.columns)
    exp ← SHAP.Explainer(XGB)
    SHAPValues ← exp(xFrame)
    SHAP.plots.bar(SHAPValues, max_display=27)
    RDS ← SelectData(SF,D)
  end for
  return RDS
end procedure=0
```

The algorithms 1 and 2 are for subsections "Dimensionality Reduction via SHAP" and "Interpretation & Dimensionality Reduction via Anchor" respectively in the main paper.

In the algorithm 1, we represent our working with SHAP for generating feature importance values using the XGBoost classifier (XGB). We take our two processed datasets, one containing suicide ideation features (Suicide_D) and the other without such features (NonSuicide_D). For each of the datasets, we perform the following steps. We split the dataset into a training set (T) and a testing set (S) using an 80-20 split ratio. The XGBoost classifier is trained on the training set (T) and used to make predictions on the testing set (S).

To calculate the feature importance, the input dataset (X) is converted into a DataFrame (xFrame) with the same column structure as the training and testing sets. Then, the SHAP explainer is created using the trained XGBoost classifier. The explainer computes the SHAP values, which quantify the contribution of each feature to the predictions made by the XGBoost model.

The computed SHAP values (SHAPValues) are then visualized using a bar plot. This plot displays the importance of each feature, ranking them based on their impact on the model's predictions. The parameter "max display=27" specifies that only the top 27 features will be shown in the plot. Finally, we create two

Algorithm 2 Algorithm to obtain top anchored features using Anchor on XGBoost

```
procedure ANCHOR(RDS)
  XGB ← XGBClassifier
  for datasets D in RDS do
    T, S ← 80_20_split(D)
    XGB ← Fit(T)
    XGB ← Pred(S)
    FeatureNames ← list(T.columns)
    df ← DataFrame(T, FeatureNames)
    exp ← AnchorTabularExplainer(T.values, FeatureNames)
    for each row r in S do
      INS ← S.iloc[r]
      P ← XGB.pred(X)
      genExp ← exp.explain_instance(P)
      record/display Anchored ← genExp.names()
      record/display Precision ← genExp.precision()
      record/display Coverage ← genExp.coverage()
    end for
    FDS ← SelectData(genExp, RDS)
  end for
  return FDS
end procedure=0
```

reduced datasets (RDS) having 27 features based on the feature importance values computed by the SHAP.

In the algorithm 2, for both of the reduced datasets (RDS) from SHAP analysis, we first split it into a training set (T) and a testing set (S) using an 80-20 split ratio. The XGBoost classifier (XGB) is then trained on the training set (T) and used to make predictions on the testing set (S).

To prepare the training set (T) for anchor explanations, we create a list of feature names (FeatureNames) from the columns of the training set. We then create a DataFrame (df) using the training set (T) and the feature names (FeatureNames). Next, an AnchorTabularExplainer is created using the values of the training set (T) and the feature names (FeatureNames). This explainer is responsible for generating anchor explanations based on the XGBoost model. We iterate over each row (r) in the testing set (S). For each row, it retrieves the instance (INS) from the testing set. The XGBoost model makes a prediction (P) on this instance. An explanation for the instance is generated using the explainer (exp) by calling the 'explain_instance()' method with the prediction (P) as input. The generated explanation (genExp) includes the anchor names, precision, and coverage.

We record and display the anchored rules, precision, and coverage for the gener-

ated explanation. This information helps to understand the rules or conditions under which the model makes its predictions. After iterating over all rows in the testing set (S), we analyze recorded generated rules and create a further reduced dataset having 12 features for suicide ideation and 19 features for without suicide ideation dataset.

Table 1: Important features for the dataset with suicidal ideation features, along with the count of how many times each feature was used as an anchor in explanations.
T - Total usage count as an anchor
$\mathbf{T^{NS}}$ - Usage count in non-suicidal class predictions
$\mathbf{T^{S}}$ - Usage count in suicidal class predictions

**Feature Name**	**T**	$\mathbf{T^{NS}}$	$\mathbf{T^{S}}$
Lifetime Suicide Attempts due to Depressive Disorders	51	43	8
Suicide Thoughts Lifetime	42	38	4
Significant Problems with Suicidal Thoughts in Life	23	22	1
Personality Disorder - Borderline	15	14	1
Times Hospitalized for Psych Problems in Life	7	3	4
Number of People Dependent Past 6 Months	3	3	0
Age of First Tobacco Use	2	1	1
Number of times Arrested while using/getting Drugs	1	1	0
Shoplifting - Lifetime	1	0	1
Cocaine Use Past 6 Months	1	1	0
Age of First Marijuana Use	1	0	1
Age of First Time in Jail	1	0	1

Table 2: Important features for the dataset without suicidal ideation features, along with the count of how many times each feature was used as an anchor in explanations.
T - Total usage count as an anchor
$\mathbf{T^{NS}}$ - Usage count in non-suicidal class predictions
$\mathbf{T^{S}}$ - Usage count in suicidal class predictions

**Feature Name**	**T**	$\mathbf{T^{NS}}$	$\mathbf{T^{S}}$
Times Hospitalized for Psych Problems in Life	54	47	7
CODSI-SMD Specificity, cut off score of 3 Severe Diagnoses Only	25	24	1
Score on 3 Item Screen for Major Disorders Only	23	19	4
Personality Disorder - Borderline	14	13	1
MHSF Total Score	14	12	2
Patient Age	5	5	0
Age of First Cocaine Use	4	1	3
Times Hospitalized	3	2	1
CODSI-SMD Specificity, cut off score of 2 Severe Diagnoses Only	3	3	0
Depressive Disorders Lifetime	2	2	0
Eating Disorders Past Month	2	1	1
Number of People Dependent Past 6 Months	2	1	1
Number of times Arrested while using/getting Drugs	1	1	0
Alcohol Type Drank Most Often Past 30 Days	1	1	0
Episodes of Major Depressive Disorders Lifetime	1	1	0
Age of First Tobacco Use	1	1	0
Total Score from TCUDS	1	1	0
Drug Use Affected Attitude/Emotional Health Past 6 Months	1	0	1
Number of times Committed Public Intoxication Past 30 Days	1	0	1

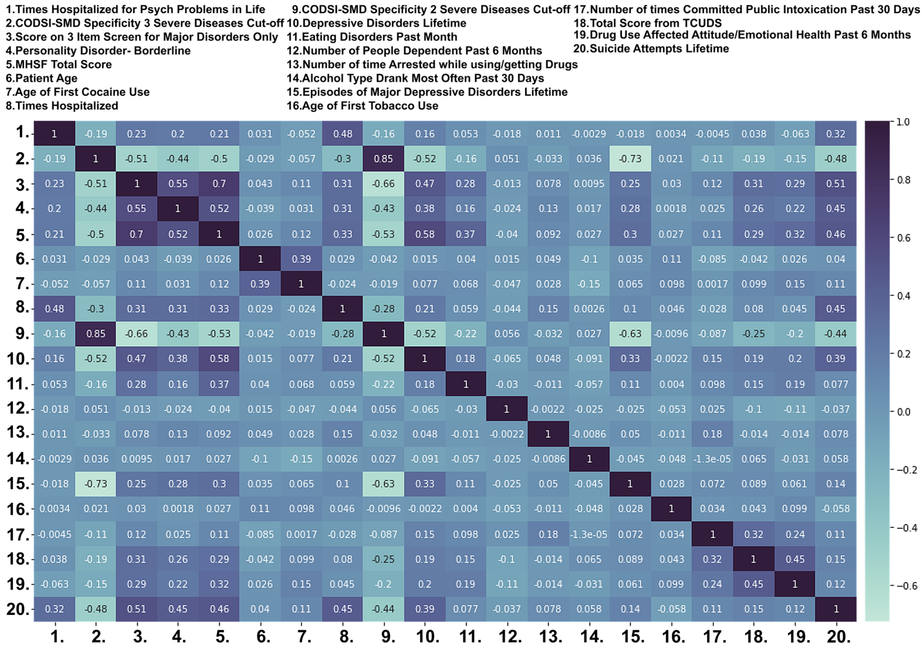

Figure 1: Pairwise correlation of anchor reduced features without suicide ideation features.

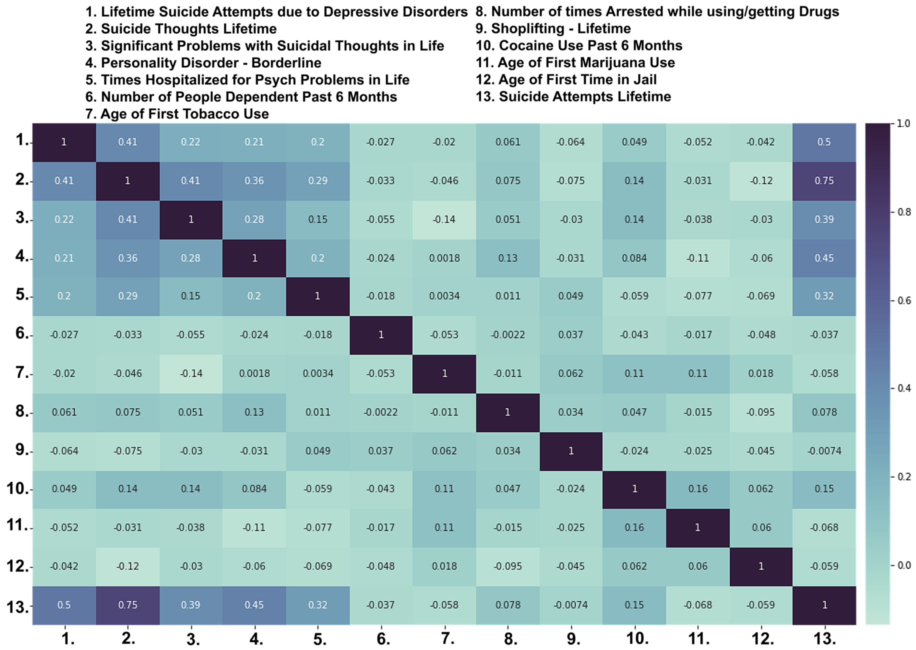

Figure 2: Pairwise correlation of anchor reduced features with suicide ideation features.

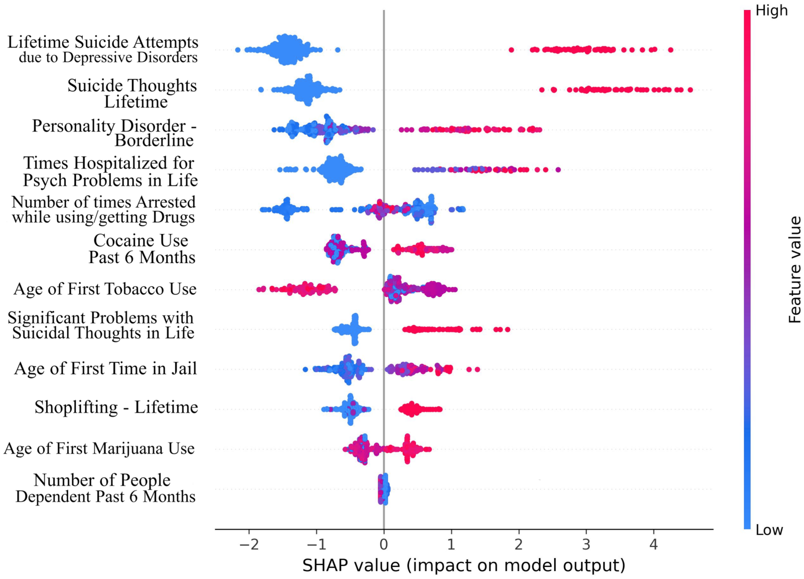

Figure 3: Feature contributions by SHAP values of anchor reduced features, including suicidal ideation features.

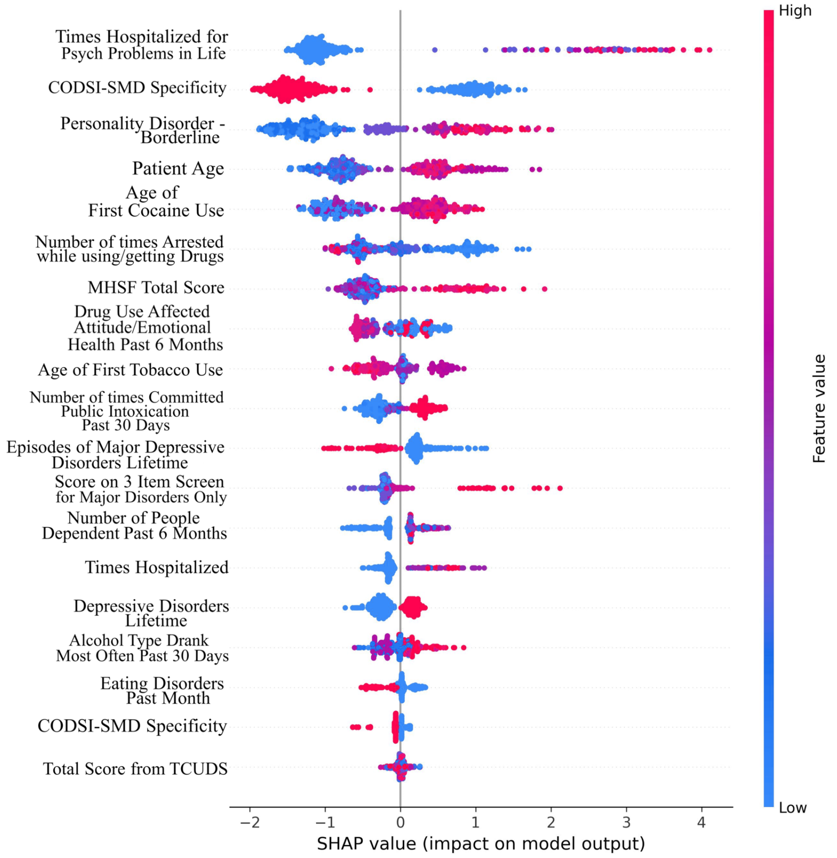

Figure 4: Feature contributions by SHAP values of anchor reduced features excluding suicidal ideation features.

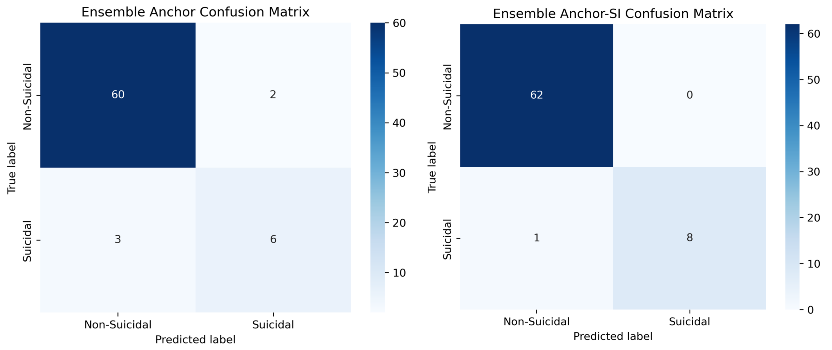

Figure 5: Confusion Matrix for Anchor reduced Ensemble model with and without Suicidal Ideation (SI) features.
